# Taro Genome Assembly and Linkage Map Reveal QTLs for Resistance to Taro Leaf Blight

**DOI:** 10.1534/g3.120.401367

**Published:** 2020-06-16

**Authors:** M. Renee Bellinger, Roshan Paudel, Steven Starnes, Lukas Kambic, Michael B. Kantar, Thomas Wolfgruber, Kurt Lamour, Scott Geib, Sheina Sim, Susan C. Miyasaka, Martin Helmkampf, Michael Shintaku

**Affiliations:** * University of Hawaii at Hilo, Department of Biology, Hilo, Hawaii,; ^†^ University of Hawaii at Manoa, Department of Tropical Plant and Soil Sciences, Honolulu, Hawaii,; ^‡^ University of Hawaii at Hilo, College of Agriculture, Forestry and Natural Resource Management, Hilo, Hawaii,; ^§^ University of Tennessee at Knoxville, Department of Entomology and Plant Pathology, Knoxville, Tennessee,; ^**^ United States Department of Agriculture-Agricultural Research Service, Hilo, Hawaii

**Keywords:** *Colocasia esculenta*, disease resistance genes, linked-read genome assembly, linkage mapping, Taro Leaf Blight

## Abstract

Taro (*Colocasia esculenta*) is a food staple widely cultivated in the humid tropics of Asia, Africa, Pacific and the Caribbean. One of the greatest threats to taro production is Taro Leaf Blight caused by the oomycete pathogen *Phytophthora colocasiae*. Here we describe a *de novo* taro genome assembly and use it to analyze sequence data from a Taro Leaf Blight resistant mapping population. The genome was assembled from linked-read sequences (10x Genomics; ∼60x coverage) and gap-filled and scaffolded with contigs assembled from Oxford Nanopore Technology long-reads and linkage map results. The haploid assembly was 2.45 Gb total, with a maximum contig length of 38 Mb and scaffold N50 of 317,420 bp. A comparison of family-level (Araceae) genome features reveals the repeat content of taro to be 82%, >3.5x greater than in great duckweed (*Spirodela polyrhiza*), 23%. Both genomes recovered a similar percent of Benchmarking Universal Single-copy Orthologs, 80% and 84%, based on a 3,236 gene database for monocot plants. A greater number of nucleotide-binding leucine-rich repeat disease resistance genes were present in genomes of taro than the duckweed, ∼391 *vs.* ∼70 (∼182 and ∼46 complete). The mapping population data revealed 16 major linkage groups with 520 markers, and 10 quantitative trait loci (QTL) significantly associated with Taro Leaf Blight disease resistance. The genome sequence of taro enhances our understanding of resistance to TLB, and provides markers that may accelerate breeding programs. This genome project may provide a template for developing genomic resources in other understudied plant species.

Taro (*Colocasia esculenta* (L.) Schott) is a food staple widely cultivated in the humid tropics of Asia, Africa, Pacific and the Caribbean. The starchy corm (underground stem) is a good source of calories, and its leaves and petioles are used as a vegetable rich in dietary fiber and vitamin C (Greenwell 1947; Rao *et al.* 2010; Kaushal *et al.* 2013). As the 5^th^ most produced root crop in the world (Food and Agriculture Organization of the United Nations 2017), taro is a vital component of many subsistence farming communities, and production issues are a serious food security concern. Taro Leaf Blight (TLB), caused by the fungus-like oomycete *Phytophthora colocasiae*, can reduce leaf yield by 95%, which in turn can reduce corm yield by up to ∼50% in susceptible cultivars (Nelson *et al.* 2011, Miyasaka *et al.* 2012, Singh *et al.* 2012). When TLB occurs as an epidemic, leaves can be destroyed in 10 days or less, leading to a decrease in photosynthesis with a subsequent reduction of corm yield (Nelson *et al.* 2011). Developing TLB-resistant varieties will improve yield, can reduce pesticide applications, and will help to ensure food security. This effort is aimed at developing a genome reference and understanding the genetic basis of resistance to TLB in taro.

The geographic center of taro domestication is not clear from archeological records, but molecular analyses indicate the presence of two separate gene pools that originate from the Indo-Malayan region in Asia and Melanesia in the Pacific (Coates *et al.* 1988, Lebot and Aradhya 1991, Kreike *et al.* 2004, Mace *et al.* 2006, Miyasaka *et al.* 2019). With a basic chromosome number of n = 14, taro occurs in diploid (2n = 28) or triploid (3n = 42) forms (Coates *et al.* 1988, Wang *et al.* 2017). Both of these forms are present in Asia, Africa, and South America, whereas triploids are generally absent from Oceania (Coates *et al.* 1988, Ochiai *et al.* 2001, Matsuda and Nawata 2002, Lebot *et al.* 2004, Chaïr *et al.* 2016). The diploid taro genome shows a wide range in estimated size, from ∼2 to ∼4 Giga base pairs [C-value mean (Bennett and Leitch 1995, Wang *et al.* 2017)], and within Micronesia and Polynesia the genetic diversity of taro cultivars is relatively low (Lebot and Aradhya 1991, Lebot *et al.* 2004). Indeed, comparisons between Papua New Guinea and Pacific Island cultivars revealed several identical genotypes between these two regions, consistent with the latter having been introduced from the former (Mace *et al.* 2006). Although genetic resistance to TLB has been identified in taro populations from Palau, Indonesia, and Papua New Guinea, all Hawaiian landraces are susceptible to TLB and many have been lost due to this disease (Miyasaka *et al.* 2012). Once established, TLB also forces costly changes to cultivation practices. Given the special cultural significance of taro in many Pacific Island societies, particularly in Hawaii (Abbott 1992, Cho *et al.* 2007, Rao *et al.* 2010), preservation of lineages is of substantial cultural and economic interest.

Taro is typically vegetatively propagated by removing and planting suckers or vegetative propagules from the tops, but hand-pollination can produce large numbers of highly heterozygous hybrids that can be selected for disease resistance and desirable agronomic traits. A collection of taro plants maintained by the University of Hawaii and used in breeding programs includes all remaining Hawaiian landraces and several cultivars from Nepal, Indonesia, Thailand, Melanesia, and Micronesia (Cho *et al.* 2007). Over the past several decades, crosses between Hawaiian and non-Hawaiian cultivars have been selected for desirable characteristics and made available for commercial planting or further breeding (Shintaku *et al.* 2016). One such cross (‘230’ x ‘255’, see methods) produced progeny that exhibited TLB resistance, but a previous linkage map developed from 240 high quality SNPs identified using a reference-free genotyping by sequencing [GBS (Elshire *et al.* 2011)] approach failed to demonstrate associations between QTL and TLB resistance (Shintaku *et al.* 2016). Thus, a high quality reference genome for improved single nucleotide polymorphism (SNP) calling and linkage mapping can serve to identify loci associated with TLB resistance and other desired agronomic qualities, and aid marker-assisted breeding programs.

Rapid advancements in sequencing technology and computer algorithms bring *de novo* assembly of high quality genomes within reach for non-model taxa, even for those with large, repetitive genomes (Goodwin *et al.* 2016, Paajanen *et al.* 2019). Genome assembly with short-read shotgun sequences carries the advantage of high sequencing depth at low per-base cost relative to long-read sequencing platforms; however, short reads are unable to traverse genomic intervals that span repetitive elements, which causes assembly fragmentation. Incorporating data from mate-pair libraries can help to reduce this fragmentation (Wetzel *et al.* 2011), but that strategy is less effective when applied to complex genomes with long [multi-kilobase (kb)] repeat elements. Alternatively, linked-reads (barcoded short-read libraries) retain long-range information while maintaining the advantages of short reads, and can produce a complete representation of the genome with high-levels of accuracy (Zheng *et al.* 2016, Weisenfeld *et al.* 2017). This genome sequencing strategy can be implemented with low DNA input, *e.g.*, ∼1 ng (Marks *et al.* 2019), and is cost-effective. Paajanen *et al.* (2019) performed a direct comparison of *de novo* genome assemblies for the potato species *Solanum verrucosum* (722 Mb genome size), and showed that a single linked-read library sequenced in a single Illumina lane (92x coverage) produced an assembly comparable in quality to an assembly reconstructed from 65 Pacific Biosystems SMRT cells (50x coverage), at a fraction of the cost (∼$4k *vs.* $25k, USA dollars). Other examples of high-quality linked-read genome assemblies for non-model species include pepper (*Capsicum annuum*) and the African wild dog (*Lycaon pictus*), and this technology been leveraged to improve genome completeness for bottlenose dolphin (*Tursiops truncatus*) and Atlantic herring (*Clupea harengus*) (Armstrong *et al.* 2018; Hulse-Kemp *et al.* 2018, Kongsstovu *et al.* 2019; Martinez-Viaud *et al.* 2019).

Coupled with a need to understand the genetic basis of TLB resistance, a taro genome reference and analysis of genotypic data from a mapping population that shows resistance to TLB infection will accelerate the understanding of the genomic underpinnings of that trait. Accordingly, the objectives of this study are to: 1) generate a *de novo* taro genome reference assembly; 2) use GBS data from a TLB-resistant mapping population to construct a linkage map and test for associations between QTL and resistance to TLB; and (3) characterize basic genome architecture of *Araceae* using available high-quality genome resource data.

## Materials And Methods

### Genome material

The Hawaiian landrace ‘Moi’ was selected for genome sequencing because it is a grandparent to ‘Parent 230’ used in the ‘1025’ TLB-resistant mapping population (see below), and because it is highly regarded for its agronomic qualities. The DNA for genome sequencing was isolated from newly emerging taro leaf tissue using a modified CTAB protocol (Healey *et al.* 2014). Briefly, ∼1 to 1.5 g of tissue was ground in 10 ml of 100 mM Tris pH 7.5, 1.5 M NaCl, 2% CTAB, and 0.3% β-mercaptoethanol, incubated for one hour at 65°, then centrifuged for 5 min at 7600 RPM to pellet cellular debris. The DNA was extracted using a standard phenol-choloroform extraction protocol with three cleaning steps, chloroform, phenol:chloroform:isoamyl alcohol (25:24:1), then chloroform, with 0.5 volume added to the supernatant in each step. After the first chloroform cleaning step the aqueous phase was treated with 5 uL RNAse A (10 mg/mL) and incubated at 42° for 15 min. All mixing was performed by gently inverting the tube for several minutes, and the aqueous phase separated from phenol/chloroform by centrifugation at 7600 RPM. The DNA was precipitated from the aqueous phase by adding 1/5 volume 5M NaCl and 1.5 volume ethanol, spooled on a heat sealed glass Pasteur pipet, and transferred to a vial containing 70% ethanol for a 10 min soak. After repeating the ethanol soak step, the spooled DNA was briefly rinsed in 100% ethanol and then allowed to air dry for several minutes. Finally, the DNA was dissolved overnight in 350 to 400 uL of TE buffer (0.2mM EDTA). The DNA was quantified with a UV spectrophotometer (Eppendorf Biophotometer), which indicated A260/A280 ratios of 1.8 to 1.9 and A260/A230 ratios between 1.9 and 2.2. The DNA concentration was determined with a Qubit fluorometric spectrophotometer and subjected to a second purification using Zymo Research Genomic Clean and Concentrator Columns using the manufacturer’s instructions.

### Sequencing library preparation

The linked-read library was prepared by the HudsonAlpha Institute using the 10x Genomics (Pleasanton, California) microfluidic gel bead partitioning Chromium system (Zheng *et al.* 2016, Weisenfeld *et al.* 2017), and was sequenced on two lanes of an Illumina HiSeq X short-read platform. The DNA for this library was prepared by performing a high-pass size-selection to collect DNA fragments >40 kb using a 0.75% agarose gel cassette and external U1 marker for the Blue Pippin system (Sage Science, Beverly, MA, USA) following manufacturer’s protocol, followed by concentration and cleaning with Ampure beads. The long-read sequences were generated on an Oxford Nanopore Technology (“nanopore”) MinION sequencing instrument using R9.4.1 chemistry (Goodwin *et al.* 2015), with sequencing conducted at the University of Hawaii at Hilo. A total of 14 FLO-MIN106R9 cells were run using standard kits and reagents and following manufacturer’s protocols. Cells routinely produced 3-5 gigabases (Gb) data, but a small portion of cells stopped prematurely [after ∼100 megabases (Mb) of data]. These failed cells were replaced by Oxford Nanopore Technology at no cost. The DNA for the nanopore long-read sequencing was sheared using Covaris G-Tubes, with most of the DNA running above 8 kb on a 0.7% agarose gel, consistent with the target fragment size.

### Genome assembly

The taro genome was assembled from linked-reads and then gap-filled and scaffolded with contiguous segments (contigs) assembled from nanopore long-reads and linkage map results. The linked-read genome was assembled with Supernova software v. 2.1.1 (Weisenfeld *et al.* 2017) by the HudsonAlpha Institute. The nanopore long-reads were assembled with Canu v. 1.7.1 and Albacore v. 2.0.1 (Koren *et al.* 2017), but those contigs provided an incomplete representation of the genome, as detailed in results, so they were used only for gap filling and scaffolding of the linked-read assembly as implemented in quickmerge (Chakraborty *et al.* 2016). That program replaces gaps present in the recipient assembly (“query”, linked-read) with sequences from the donor assembly (“reference”, nanopore), discarding donor contigs without match to the query. The resultant gap-filled, linked-read assembly is hereafter referred to as the “merged” genome. For contig alignment, quickmerge implements MUMmer (Delcher *et al.* 2003, Kurtz *et al.* 2004) programs nucmer and delta-filter, with a threshold cut-off of 95% sequence similarity. To quantify similarity between assembled contigs of linked-read and nanopore assemblies those were pairwise aligned and assessed with MUMmer programs nucmer and dnadiff (Delcher *et al.* 2003, Kurtz *et al.* 2004), with settings delta-filters 1-to-1 alignment and minimum alignment length of 10 kb. Excepting the linked-read assembly, all analyses were performed on the University of Hawaii High Performance Computing cluster (server size: 20 core, 120 gigabyte RAM). Programs were run using default parameters unless otherwise noted above.

The haploid genome (1C) size of ‘Moi’ was estimated to be 2.39 Gb, based on the read k-mer profile analysis conducted in Supernova (Weisenfeld *et al.* 2017). The data inputs for the linked-read genome included 580 million linked-reads of mean length 150 bp (∼174 Gb), of which 89% were in proper read pairs. The nanopore MinION cells produced a total of 12.3 million long-reads, for a total of 63.7 Gb. For descriptive purposes, sequence data from one to three MinION cells were combined into a total of 11 groups, with summary statistics provided in Supplemental Table 1. To reduce computational demand during long-read contig assembly we discarded short long-reads (< 3.5 kb length, n = 6.5 million) and outlier long-reads (> 150,00 kb length, n = 43), thus retaining 5.8 million long-reads totaling ∼54 Gb. Accounting for the total numbers of sequenced nucleotide bases, read coverages for the linked- and long-read assembles were ∼60x and ∼23x, respectively.

### Genome assembly characterization, quality metrics, and filtering

The linked-read and merged assemblies were quality assessed using descriptive measures, *e.g.*, numbers of contigs, total number assembled bases, and completeness, implementing analyses tools QUAST (Gurevich *et al.* 2013) and the Benchmarking Universal Single-Copy Orthologs pipeline [BUSCO v4.0.5 (Seppey *et al.* 2019)]. The latter accessed programs Augustus v3.2.3, Blast+ v2.2.31, and HMMER v3.2., and utilized the OrthoDB plant database liliopsida_odb10 for class monocot containing 3,236 single-copy genes from 10 species (Kriventseva *et al.* 2019). For comparative purposes BUSCOs were also analyzed for genomes of seven other monocots spanning five orders. These included: Asparagales, asparagus (*Asparagus officinalis*, GCF_001876935) and orchid (*Phalaenopsis equestris*, GCF_001263595.1); Arecales, coconut (*Cocos nucifera*, GCA_008124465.1*)*; Dioscoreales, trifoliate yam (*Dioscorea dumetorum*, GCA_902712375.1); Poales, sorghum (*Sorghum bicolor*, GCF_000003195.3); and Alismatales, eelgrass (*Zostera marina*, GCA_001185155.1) and great duckweed (*Spirodela polyrhiza*, GCA_001981405.1). The GCF’s and GCA’s refer to National Center for Biotechnology Information (NCBI) genome accession identifiers. Taro and duckweed are members of the *Araceae* family, but duckweed has a much smaller genome size (158 Mb) and a high-quality, chromosome-level genome assembly (20 chromosomes, N50 7.6 Mb) has been produced (Michael *et al.* 2017). A second BUSCO analysis was performed for taro only using the eukaryota_odb10 eukaryote database that contains 255 genes from 70 species.

To prepare for scaffolding with the linkage map, the merged assembly was filtered for assembly artifacts by removing: 1) stretches of Ns at the beginning and ends of scaffolds, 2) scaffolds consisting entirely of Ns, 3) duplicate scaffolds identified by the MUMmer (Delcher *et al.* 2003, Kurtz *et al.* 2004) package dnadiff or flagged by NCBI’s genome filtering process, and 4) contigs/scaffolds containing potential contaminants. The latter were defined as producing blastn hits against 15,180 records of complete bacterial genomes available through NCBI’s FTP site (accessed October 30, 2019), meeting the following cut-offs: 90% identity, length >100 nucleotides, and with match over >10% total contig length. Finally, the merged, filtered assembly was scaffolded using the linkage map (see below) to produce a pseudochromosome-level assembly.

### Genome architecture

To gain insights into genome architecture of Araceae, the genomes of taro and great duckweed were assessed for repetitive content and identification of gene domains characteristic of nucleotide-binding leucine-rich repeat (NLR) plant disease resistance genes. These genes also occur in animals, and are crucial regulators of inflammatory and innate immune responses. The repetitive content was characterized by generating *de novo* repeat libraries with RepeatModeler v1.0.11 (Smit and Hubley 2008-2015), which finds interspersed repeats by integrating RepeatScout v1.0.05 (Price *et al.* 2005) and RECON v. 1.08 (Bao and Eddy 2002). Based on each assembly’s custom repeat library, interspersed repeats, simple repeats, and low complexity regions were quantified using the quick search option of RepeatMasker version open-4-0-9-p2 (Smit *et al.* 2013-2015) run with RMBlastN 2.9.0+-p1 and the RepeatMasker combined database Dfam 3 [(Hubley *et al.* 2016) download date 8-25-2019]. A second assessment of repeat content was constructed from all plant repeat databases available in RepeatMasker, including species databases monocotyledons (liliopsida), arabidopsis, rice, wheat, and maize, but those analyses were discarded after proving to be less sensitive than our custom repeat libraries. Relevant to TLB-resistance, the complement of NLR sequences was quantified with NLR-Annotator v0.7-beta (Steuernagel *et al.* 2015) using motifs defined by Jupe *et al.* (Jupe *et al.* 2012). NLR-Annotator identifies domains characteristic of the NLR gene family, and returns sequences from the start of the first domain, but not the start to end of the entire protein sequence. The advantage to this approach is that it is unbiased by protein prediction models. The great duckweed genome was included in the NLR analysis for comparative purposes.

### TLB-resistant crosses and phenotypic analysis

The TLB mapping population ‘1025’ was created by crossing two breeding lines ‘230’ and ‘255’ (see also Shintaku *et al.* 2016). Parent ‘230’ is a cross between Hawaiian landrace ‘Moi’ × TLB-resistant Palauan landrace ‘Dirratengadik’, and Parent ‘255’ is a hybrid between ‘Sawahn Kurasae’ (TLB-resistant landrace from Indonesia) and ‘RMP-08’. ‘RMP-08’ is itself a hybrid between the Hawaiian ‘Red Moi’ and the Papua New Guinea ‘PH15’ cultivar. More than 2500 plants representing parents and progeny from 27 crosses were screened using a modified detached leaf disk assay (Brooks 2008) to define this particular TLB-resistant mapping population (Shintaku *et al.* 2016), crosses were repeated, and TLB resistance in several cultivars was confirmed by field tests. The ‘1025’ mapping population was challenged with *P. colocasiae* isolates ‘S1’ and ‘S3′ collected from taro fields at Pepeekeo and Panaewa, Hawaii, respectively. Pure *P. colocasiae* cultures were established on a base of 10% V8 media plated into a petri dish (50 ml V8, 0.624 g CaCO_3_, 450 ml water, 10g agar) overlaid with 1.5% water agar plugs cut into a circle ∼0.5 cm in diameter, that was in turn overlaid with agar plugs from colonized plates. After establishment, cultures were grown by incubating on 10% V8 media at 27**°** for 5 to 10 days to obtain zoospores. The zoospores were collected from each culture plate by flooding with 10 ml of sterile distilled water followed by incubation at 4° for 30 min. The plate was then left for 20 to 25 min to adjust to room temperature, and then swirled gently and the water pipetted off and placed in a tube to count zoospores on a hemocytometer. The spore suspension was diluted to approximately 30 zoospores per microliter.

For disease challenges, 24 mm diameter leaf blade discs were cut from the ‘1025’ mapping population using a cork borer. The second leaf blade from each plant (where leaf blade one is the first fully matured leaf blade) was used in the assay. Leaf discs were placed on 0.9% agar with the adaxial surface exposed. Four leaf blade discs from the same genotype were assayed in separate Petri dishes. In each assay, taro variety ‘Bun Long’ was used as a susceptible check cultivar. Leaf discs were inoculated with 10 µl water containing ∼300 *P. colocasiae* zoospores. The lesion diameter of the four discs was standardized to that of the susceptible cultivar. As an example, a relative lesion size of 0.5 indicates that the individual has half of the lesion diameter of the susceptible check cultivar.

### Genotyping materials and SNP calling

The TLB-resistant mapping population consisting of Parents ‘230’ and ‘255’ and 92 of their progeny were sequenced using GBS (Shintaku *et al.* 2016) (NCBI SRX2754312), calling SNPs with the merged genome as a reference. To improve confidence in SNP calls, we included an additional taro GBS dataset comprised of 95 taro accessions that originated from Hawaii, South Pacific, Palau, and mainland Asia (Helmkampf *et al.* 2018; NCBI SRX2754311). This raised the total number of GBS samples to 189. For GBS, genomic DNA was isolated from freeze-dried or fresh leaf tissue using the Macherey-Nagel Nucleospin Plant II kit or Qiagen Plant DNeasy Mini kit according to each manufacturer’s protocol. The GBS libraries were digested with the restriction enzyme *PstI* and prepared for sequencing at the Cornell University Genomic Diversity Facility (Ithaca, NY). Each set of samples was sequenced on a single flow cell lane of an Illumina HiSeq 2500 using single-end protocols to produce reads of length 100 bp. The GBS data were demultiplexed and barcodes trimmed using software GBSX v1.1.4 (Herten *et al.* 2015). Across all 189 GBS samples approximately 459 million usable reads were obtained. The Illumina sequences were mapped to the reference using Bowtie2 v2.2.4 (Langmead and Salzberg 2012) with setting *–very-sensitive-local*. Variants were called from the output of SAMtools v1.4.1 mpileup using the bcftools v1.2 multiallelic calling model (Li *et al.* 2009; Danecek *et al.* 2016), considering only uniquely mapped reads with threshold map and base quality phred quality scores of 20. After removing non-target samples, the parent and progeny variant call format (VCF) files were preliminarily filtered by discarding SNPs within 5 base pairs of an insertion/deletion (INDEL) site, removing INDELS, and removing samples with high levels of missing data (n = 8). Next, VCF files were iteratively filtered using VCFtools v0.1.14 (Danecek *et al.* 2011) to retain di-allelic SNPs with a minimum sequencing depth of at least eight reads per genotype, a per-locus data density of minimum 80%, and setting the minor allele frequency (maf) threshold to 0.012. Loci identified as invariant in the parents were removed, with allowance for missing data in one but not both parents. In preparation for linkage mapping and QTL analyses, the parental genotypes were phased and progeny genotypes imputed in beagle v5.0 (download date 16May19.351; Browning and Browning 2009).

### Linkage map and QTL analysis

A linkage map was constructed using GBS data from 84 individuals of the ‘1025’ mapping population that passed quality controls. The variant calls (SNPs) were filtered for segregation distortion, redundancy, homozygous condition in parents (genetically plausible because the ‘230’ and ‘255’ parental stocks consisted of multiple lines of clonal propagules), and unlinked loci. We aimed for 14 linkage groups, the number of haploid chromosomes in taro, and define “major linkage group” as having >8 markers per group. The grouping of SNP markers was performed using the OneMap package (Margarido *et al.* 2007) in Rstudio v1.1.423 (RStudio Team 2015) with R v3.5.0 (R Core Team 2019). Markers were anchored to their respective groups based on the OneMap output, and linkage maps were constructed using the CDM functionality of software package Genetic Analysis of Clonal F1 and Double cross populations (GACD) (Zhang *et al.* 2015) with setting linkage phases originally unknown. The Kosambi mapping function was used to convert the recombination frequency to map distance in centimorgans (cM) (Kosambi 2016). Software suggested logarithm of the odds (LOD) scores were used to construct and assign markers to different linkage groups (LGs), with a LOD score of 5.97 used for the final linkage map. The GACD software was also used to identify regions in the taro genome that correlated with the TLB resistance in the mapping population, with the mapping algorithm Inclusive Composite Interval Mapping of Additive and Dominant QTL (ICIM - ADD). ICIM is suitable for mapping a small population size as it controls for the bias due to the Beavis effect—*i.e.*, the overestimation of explained phenotypic variance in small populations (Xu 2003). The LOD threshold was calculated by 1000 permutation tests at α = 0.05. The QTL mode of action (*i.e.*, selection model) was calculated using the method of Muchero (Muchero *et al.* 2013),

a={mu(ac)−mu(bd)}/2;

d={mu(ad)+mu(bc)}/2−{mu(ac)+mu(bd)}/2

where ‘a’ and ‘d’ are the additive and dominance effects respectively. The *mu*(*ac*) and *mu*(*bd*) are the phenotypic means for the heterozygous loci having alleles from the same species, and *mu*(*ad*) and *mu*(*bc*) are the phenotypic means for the heterozygous loci carrying alleles from both species. The ratio of d/a is used to assess the QTL mode of action: a d/a ratio of <1 indicates underdominance, ratio between 0 and 1 indicates partial dominance, and a ratio of >1 indicates over-dominance (Muchero *et al.* 2013).

Using the final linkage map as a guide, contigs were anchored and concatenated into pseudochromosomes, orienting each contig to the linkage map position starting from marker position 0 and maintaining directionality of contigs when >1 markers per linkage group corresponded to >1 SNP loci per contig. The correspondence between markers assigned to linkage groups and SNP positions on the linkage map was further applied to assign confidence rankings using a 4 point scale. A high-confidence score of 1 was assigned when markers in a linkage group were consistently ordered with SNPS on single contigs and separated by a reasonable distance, defined as exceeding that of the median sequence length used to generate the assembly. A score of 2 was assigned to markers that corresponded to a single SNP on a single contig, and a score of 3 was assigned to markers that corresponded to clustered SNPS (defined as < median sequence length of the assembly), or were ordered discordant from the linkage group map distance. Last, loci from single contigs assigning to >1 major linkage groups were scored as 4. These contigs were included in each LG pseudochromosome, therefore a small portion of the linkage groups contain redundant sequences. Only markers of score of 1 were used to assign contig directionality during contig concatenation into pseudochromosomes.

### Data availability

The pseudochromosome-level reference genome assembly has been deposited at DDBJ/ENA/GenBank under the accession WUBK00000000, BioProject PRJNA567267. The version described in this paper is version WUBK01000000. File S1 contains detailed descriptions of supplemental results (Tables, Figures, and Appendixes), and documents archived in figshare (linked-read and merged draft genomes, nanopore contigs, the vcf file used for linkage mapping and QTL analysis, the R script used to construct linkage groups, phenotypic data for the mapping population, and the *de novo* repeat library from RepeatModeler). The raw Illumina sequences and corresponding 10x Genomics barcode files are available through the NCBI Sequence Read Archive (SRA) SRP223785 and SRS5458621, and the Oxford Nanopore Technology long-read sequencing files (prior to self-correction) are available through the BioProject ID PRJNA567267. The previously published GBS datasets analyzed in this study were obtained from the NCBI SRA database under BioProject PRJNA381383 (Accessions: SRX2754311-12). The SNP calling and filtering pipeline is available online (Bellinger 2019). Supplemental material available at figshare: https://doi.org/10.25387/g3.12440039

## Results

### Genome assembly characterization and quality metrics

The pseudochromosome-level taro genome is estimated to be mostly complete, with the total number of assembled bases, ∼2.45 Gb, slightly larger than the k-mer estimated genome size of 2.39 Gb. The assembly’s largest scaffold was 38 Mb, and after excluding a large number of short contigs (< 5 kb), consists of 55,692 scaffolds representing 2.24 Gb (Table 1). The quickmerge step did little to improve genome assembly quality, decreasing fragmentation by only ∼2k contigs and increasing assembly size by only 119.8 Mb. That low amount of gap-filling can be explained by the majority of nanopore contigs failing to align to the linked-read assembly. While nanopore long-read sequencing resulted in 1.44 giga assembled bases (72,696 contigs), only 602 Mb (< 42%) of those assembled bases aligned to the linked-read genome assembly at the 95% identity threshold. In Table 1, the slight difference in number of assembled bases between the merged and pseudochromosome-level assemblies is because numbers are reported for the unfiltered merged genome, to maintain direct comparability with the (unfiltered) linked-read genome.

**Table 1:** Descriptive characteristics for three draft taro genome assemblies. The pseudochromosome-level taro assembly (“Ps_chr”) was composed from a linked-read assembly (“LR”) that was gap-filled using contigs assembled from nanopore MinION long-reads (merge step, “Merged”), filtered for assembly artifacts, and then concatenated into pseudochromosomes using a linkage map. Kilobase = kb

			# Scaffolds (% of assembled nucleotides)					
	Assembled bases	Largest scaffold	> 5 kb	> 10 kb	> 50 kb	Total	N50	GC content (%)
Ps_chr	2,448,853,100	38,380,923	55,692 (92%)	25,535 (83%)	5,180 (68%)	140,400 (100%)	317,420	41.98
Merged	2,451,639,670	6,251,291	56,504 (92%)	25,535 (83%)	5,527 (67%)	142,854 (100%)	270,514	42.04
LR	2,331,885,920	3,965,393	53,768 (91%)	22,039 (81%)	4,782 (68%)	144,852 (100%)	336,981	42.13

**Figure 1:**
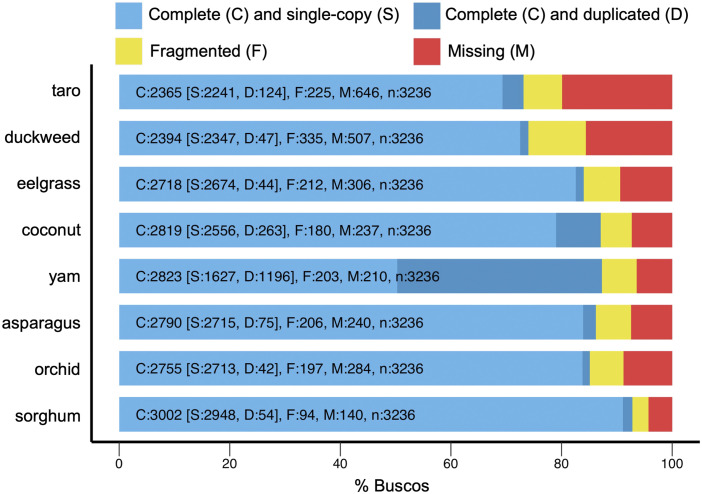
Genome assembly completeness assessed by the recovery of Benchmarking Universal Single-Copy Orthologs (BUSCOs). The percent of BUSCO genes found in each genome is listed for categories single (S) or multiple copies (D), as well as fragmented (F) and missing (M). Analyses are based on the BUSCO liliopsida_odb10 dataset representing class monocot (n = 3,236 genes). See text for scientific names and NCBI genome accession identifiers.

Based on the benchmarking gene set for monocots, completeness of the taro genome assembly is close to the high-quality, chromosome-level duckweed genome assembly, with overall recoveries of 80% and 84% BUSCOs (Figure 1). The proportion of recovered complete and single copy BUSCOs was nearly identical for the two species, 73% and 74%. Turning focus to the percent of complete and duplicated BUSCOs, the value of 3.4% for taro genome was only slightly higher than the value of 1.5% for the duckweed genome. As an outlier, the genome assembly of yam, sequenced using nanopore technology, showed a disproportionate number of duplicated benchmarking genes. Compared to the phylogenetically diverse group of monocots included in our analysis, taro and duckweed exhibited a higher fraction of missing or fragmented genes, with 43% of the missing or fragmented genes of taro also fragmented or missing in duckweed. Given the high-quality of the great duckweed genome, this finding suggests that members of Araceae differ from other monocots by types of gene losses. The BUSCO recovery rates for the non-Araceae monocots were similar regardless of whether their genomes were represented in the liliopsida_obd10 reference gene set (asparagus, eelgrass, orchid, and sorghum). Although results based on the eukaryota_odb10 benchmarking gene set shows taro to have a greater BUSCO recovery rate, encompassing 88.2% genes complete (79.2% single copy; 9.0% duplicated), 5.1% fragmented, and 6.7% missing, that assessment is less comprehensive because that database contains substantially fewer genes than the benchmarking gene database for monocots.

### Genome architecture

*De novo* repeat modeling revealed that the genome of taro is highly repetitive, with RepeatMasker screening estimating that 80% is comprised of mobile genetic elements (Table 2). Comparing the genome of taro to that of great duckweed indicated that the latter had substantially lower mobile genetic element content, 20%. The proportion of long terminal repeat (LTR) elements in taro and duckweed genomes, ∼36% and 9%, was proportionally similar to their unclassified repeat content, ∼38% and 11%. The genomic proportion of long interspersed elements (LINEs) was low across both taxa (<=1.6%), with only LINE1 types detected in the custom repeat libraries. Short interspersed element (SINEs) were detected only in the duckweed genome. The simple repeat and low complexity contents were also low in both taxa, <= 2.3%. Cumulatively, the total repeat content of taro and duckweed genomes was ∼82% and 23%, respectively.

**Table 2:** Repetitive content of taro (*Colocasia esculenta*) and great duckweed (*Spirodela polyrhiza*) genome assembles. Total repeat content was quantified using *de novo* repeat libraries constructed with RepeatModeler and screened with RepeatMasker. The percent (%) of sequence is relative to each individual assembly’s total length excluding runs of NNN”s between scaffolded contigs. Short and long interspersed elements are denoted as SINEs and LINEs

Element	Length Occupied (bp)		% Genome	
	taro	duckweed	taro	duckweed
SINEs	0	2,139	0.0	<0.01
LINEs	36,753,946	366,596	1.6	0.3
LINE1	33,001,257	366,596	1.4	0.3
LTR elements	834,904,293	12,263,206	36.3	9.1
DNA elements	91,787,447	221,956	4.0	0.2
Unclassified	874,609,262	14,284,860	38.1	10.6
Total interspersed repeats	1,838,054,948	27,138,757	80.0	20.2
Simple repeats	35,678,844	3,145,222	1.6	2.3
Low complexity repeats	8,251,536	671,992	0.4	0.5
Total assembly L	2,451,787,670	135,172,123		
L excluding Ns runs	2,297,336,160	134,368,765		
Bases masked	1,881,985,328	30,955,971		
Total % repeat content			81.9	23.0

Analysis of taro with NLR-annotator identified 391 NLRs, of which 182 were categorized as complete, 109 complete but pseudogenes, and a further 80 annotated as partial only, which indicates a lack of some NLR specific domains (Supplemental Appendix 1). In contrast, the great duckweed genome was estimated to contain only 70 NLRs, of which 46 were categorized as complete, 12 partial, and 12 complete pseudogenes. Members of the NLR gene family divide into two groups, Toll and human interleukin-1 receptor (TIR) proteins (known as TIR-NB-LRR or TNLs) and the non-TIR class of Nucleotide-binding site leucine-rich repeat proteins known as CNLs (also known as CC-NBS-LRR) because some (not all) contain a coiled-coil (CC) structure in the N-terminus domain (Jupe *et al.* 2012). Among the taro NLRs we detected all were characterized as belonging to the non-TIR class, CNL, with only six exceptions. Those six TNLs were evenly split among categories complete, partial, and pseudogenes (one complete and one partial). The great duckweed genome contained only CNLs, with the exception of one TNL annotated as a pseudogene.

### GBS data and genetic linkage mapping

**Figure 2:**
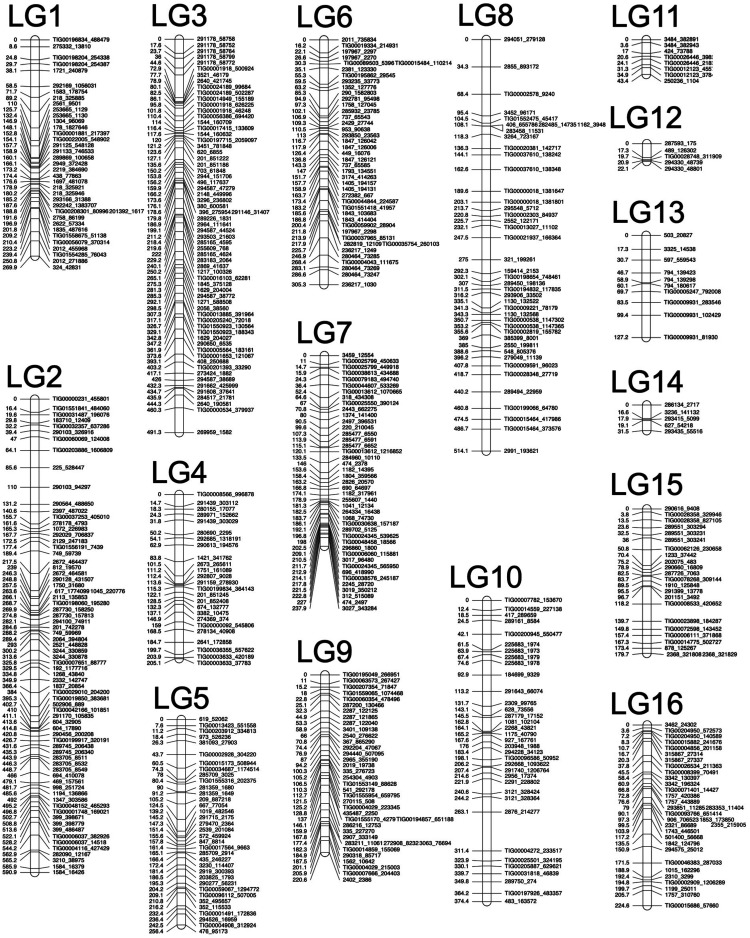
Genetic map of taro based on 520 high quality SNP markers covering 16 linkage groups.

The per-sample average number of GBS reads was ∼2.26 million, of which ∼1.5 million mapped to exactly one genome location (Table 3). From the 1,519 filtered SNPs calls using those mapping data, 1,423 passed the initial test for segregation distortion, but 865 of those markers were discarded because of homozygosity in both parents (n = 107), redundancy (n = 514), deviance from Mendelian segregation ratio (n = 179) or unlinked status (n = 65) (Table 4). Accordingly, 558 (39.21% of 1,423) SNP markers were assigned to 31 linkage groups, which equates to a total length of ∼285 Mb and represents 11.64% of the genome (Table 5). Taro has a haploid chromosome of x = 14: in our analysis only 14 linkage groups had number of markers > 8 and there was a heavy reduction in the number of markers assigned to linkage groups 17 through linkage group 31 (Supplemental Figure 1). Thus, we included the first 16 linkage groups for linkage map development and QTL analysis. The final linkage map has a total distance of 4094.38 cM, 16 linkage groups, n = 558 markers, and covers a total of 285,477,188 nucleotides (10.83% of the genome; Figure 2 and Table 6). Concatenating contigs into pseudochromosomes based on linkage groups and marker scores (Supplemental Appendix 2), along with artifact filtering, reduced the number of contigs to 140,400, as reported in Table 1.

**Table 3:** Genotyping by sequencing (GBS) mapping data and variant calls for 86 taro samples. Summary data include sample-averaged counts and percentages for total mapped and unmapped reads and reads that mapped uniquely (1x) or were multi-mapped (>1x). Numbers for variant calls include raw variable sites, INDELs, variable sites that passed filters (see text), and the number of variable sites present the mapping population (MP) after applying a minor allele frequency (maf) threshold of 0.012

Numbers of reads (%)			Mapped (%)		Numbers of variant sites			
Total	Mapped	Unmapped	1x	>1 x	Raw data	INDELs	Filtered	MP
2,723,124	2,257,353 (82.9%)	465,771 (17.1%)	1,502,585 (66.4%)	754,767 (33.6%)	15,021,591	10,982	7,018	1,519

**Table 4:** Linkage mapping results for GBS data mapped to the merged taro genome reference. Markers passing the initial test for segregation distortion are listed as number of initial markers. Homozygous, redundant, and distorted SNPs were removed from the subsequent analysis. After binning of unique markers and filtering for segregation distortion, a suggested logarithm of the odds (LOD) score was calculated and used for linkage group (LG) formation

# Initial markers	# Homozygous markers	# Unique bins	% distorted markers	Suggested LOD	# LG	# markers final map	# LG >= 8 markers
1,423	107	802	22%	5.97	31	558	14

**Table 5:** Descriptors for linkage groups (LG) constructed from ‘1025’ taro mapping population genotypes. Sixteen major linkage groups (LG) were present in the final linkage map constructed from a mapping population of taro. The SNP markers were called using the “merged” taro genome assembly as a reference (see text for details)

Linkage groups	Total markers	LG length (cM)	Average distance (cM)	Genetic length
LG1	37	269.93	7.3	24,124,590
LG2	73	590.87	8.1	42,680,486
LG3	65	491.26	7.6	30,369,817
LG4	25	205.08	8.2	16,485,140
LG5	35	256.4	7.3	18,072,045
LG6	43	305.31	7.1	13,916,264
LG7	43	237.94	5.5	18,481,439
LG8	43	514.12	12	23,744,905
LG9	38	220.57	5.8	21,816,219
LG10	35	374.35	10.7	15,471,071
LG11	8	43.42	5.4	4,286,715
LG12	5	22.09	4.4	2,732,664
LG13	10	127.25	12.7	4,820,723
LG14	5	31.51	6.3	621,528
LG15	23	179.72	7.8	11,651,500
LG16	32	224.56	7	16,237,138
Total	520	4,094.38	123.3	265,512,244
Average	33	255.90	7.7	16,594,515

**Table 6:** A list of QTL significant for TLB resistance in the ‘1025’ TLB-resistant mapping population. The QTL naming convention includes the isolate name preceded by a q, DPI4, the linkage group identifier, and a number representing the number of significant QTL in the linkage group. PVE = Phenotypic variance explained, a = additive effect, d = dominance effect, d/a = QTL mode of action

QTL name	Isolate	LG	Left Marker	Right Marker	LOD	PVE (%)	a	d	d/a
qS1_DPI4-3-1	S1	3	TIG00056386_694420	1544_160709	22.38	10.25	0.03	0.46	16.17
qS1_DPI4-6-1	S1	6	TIG00044844_224587	TIG01551418_41957	29.56	17.52	0.29	0.02	0.06
qS1_DPI4-8-1	S1	8	3452_96171	TIG01552475_45417	25.74	13.64	0.29	0.00	0.00
qS1_DPI4-8-2	S1	8	TIG00020381_142717	TIG00037610_138242	19.66	8.82	0.00	0.43	∞
qS1_DPI4-9-1	S1	9	TIG01555954_659795	270115_508	22.68	10.74	0.25	−0.02	−0.09
qS3_DPI4-2-1	S3	2	399_398671	399_398779	7.70	7.75	−0.16	−0.04	0.23
qS3_DPI4-3-1	S3	3	293503_21603	285165_4595	9.60	9.97	0.24	−0.06	−0.24
qS3_DPI4-3-2	S3	3	285165_4624	283183_2064	19.66	27.86	−0.34	−0.05	0.15
qS3_DPI4-6-1	S3	6	TIG00044844_224587	TIG01551418_41957	9.81	9.85	0.24	−0.02	−0.08
qS3_DPI4-7-1	S3	7	1068_74730	TIG00030638_157187	6.75	6.55	−0.03	0.32	−9.81

### QTL analysis based on the genetic map

**Figure 3:**
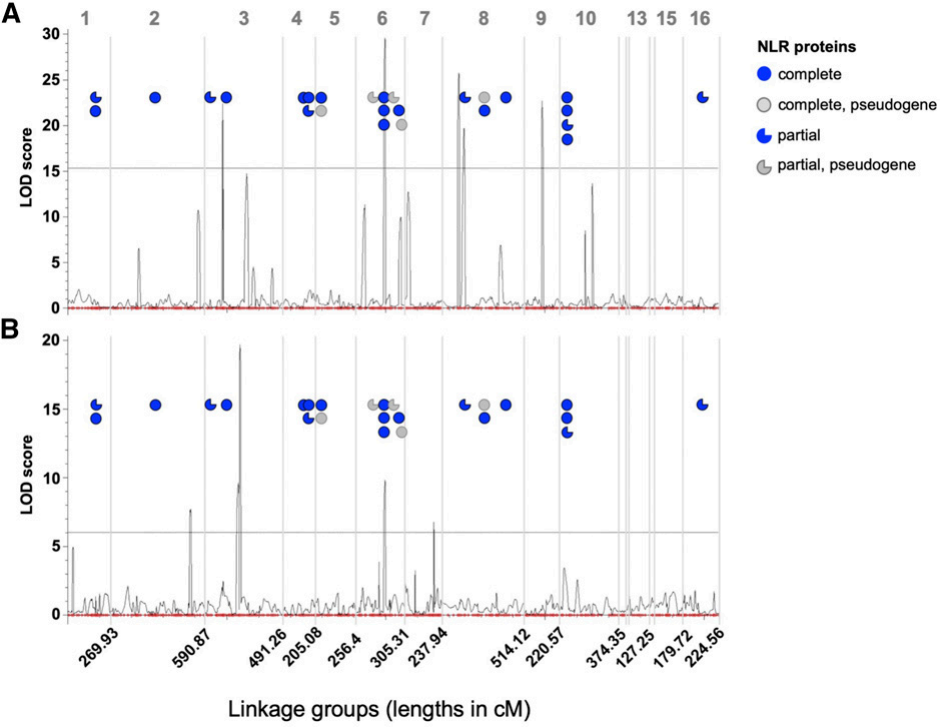
Genome wide representation of major linkage groups, including significant QTL and their LOD scores for S1 (A) and S3 (B) isolates of *Phytophthora colocasiae* exposed to the ‘1025’ mapping population. The Y-axis indicates the logarithm of odds (LOD) values. Peaks above the threshold (dotted line) of LOD = 15.32 (S1 isolate) and LOD = 6.01 (S3 isolate) represent a QTL having significant interaction with the TLB tolerance. Linkage groups >100 centimorgans (cM) are labeled, with total length shown on y-axis. Approximate locations of nucleotide binding leucine rich proteins (NLRs) (complete, partial, and pseudogenes) are indicated by colored symbols.

Composite interval mapping identified a total of 10 QTL associated with taro’s resistance to two isolates of *P. colocasiae* (Table 6). Eight QTL (four per isolate) were unique, whereas one QTL mapped to the same interval (LG6, TIG00044844_224587 and TIG01551418_41957) in both isolates, explaining 17.52% and 9.81% of phenotypic variation for S1 and S3 isolates respectively (Table 6, Figure 3). In terms of genetic selection models, the mode of action for this QTL differed between the two isolates: partial dominance was indicated for the S1 isolate, while underdominance was indicated the S3 isolate. Two QTL showed evidence of overdominance (d > 1), and of the remaining six QTL (with d < 1), three showed partial dominance and three showed underdominance, as indicated by positive or negative values of d in Table 6. The locations of QTL significant for resistance to TLB respective to all markers on LGs are shown in Supplemental Figure 2.

Among the total complement of sequences containing NLR domains, 25 complete NLRs were located within sequences assigned to LGs and tested for QTL association with TLB resistance. Although none of the NLR domains were contained within those QTL, some were within close proximity. A cluster of three NLRs (1405_nlr_1, 1405_nlr_2, and 1405_nlr_3) occurred on LG6, near the QTL associated with resistance to both S1 and S3 *P. colocasiae* isolates (Figure 3). These NLRs were characterized as CNLs, and contained a pre-NB domain. The highest concentration (n = 9) of complete NLRs occurred on LG18, representing 5% of taro’s genome-wide NLR complement, yet no QTL significant for association with TLB mapped to that particular LG. Among the nine partial NLRs that mapped to LGs, only one (NLR, 406_nlr_1) was proximate to a QTL, in this case mapping to LG8 in the interval between QTL qS1_DPI4-8-1 and qS1_DPI4-8-2.

## Discussion

We sequenced and assembled a taro genome using a linked-read sequencing strategy, with genome contiguity improved through gap filling and scaffolding using contigs assembled from Oxford Nanopore MinIon long-reads and linkage map results from a mapping population for TLB-resistance. By constructing a pseudochromosome-level reference genome, we provide a foundational resource for identifying genomic elements important for agronomic traits and resistance to disease. This draft taro genome is mostly complete, based on the total number of assembled base pairs compared to the k-mer estimated genome size and BUSCO results. Efforts for assembly improvement can focus on increasing contiguity through application of longer-range scaffolding data such as *in vitro* Hi-C (Dovetail) or optical mapping (BioNano Genomics).

For large, highly repetitive genomes, assembly with noisy long-reads may be particularly challenging because of the requirement to disambiguate highly-similar, but non-homologous sequences. Assembling mobile repetitive elements requires sequencing through entire repeat elements that can exceed long-read lengths. For example, a survey of 50 plant genomes indicated that retrotransposons averaged 8,611 bp (Ou and Jiang 2018) in length, and can exceed >30 kb (Xu *et al.* 2010, Ma *et al.* 2019). In this study nanopore contigs were assembled from nanopore long-reads using Canu (Koren *et al.* 2017), which includes a read error correction step, yet sequencing depth may have been insufficient to achieve the levels of self-correction necessary to assemble homologous sequence components. Systematic error in raw nanopore sequencing data are known to lead to reduced accuracy of Canu-corrected reads, for example, at theoretical 30x coverage reads averaged 92% identity to a human genome reference after correction (Jain *et al.* 2018). Our long-read coverage at ∼23x recovered 1,44 Mb of taro genome sequence (60% of the estimated genome size), but only 602 Mb (< 42%) of those assembled bases aligned to the linked-read genome assembly (at the 95% identity threshold). Instead of collecting additional long-read data, we opted to generate a new linked-reads assembly, restricting use of the nanopore contigs for gap-filling and scaffolding only. Our results show that genome contiguity was only slightly improved by that gap-filling (merge) step, based on N50 values. We note here that nanopore genome polishing with Illumina short-read data only–*e.g.*, nancorr and MaSurCa approaches (Zimin *et al.* 2013, Goodwin *et al.* 2015)–would have been a far less effective approach for genome improvement, because short reads cannot polish regions that have ambiguous mappings, as occurs for repetitive regions (Jain *et al.* 2018). In today’s dollars the nanopore contigs were sequenced using a 12-cell kit that, with reagents, cost ∼$12,500 USA dollars, in contrast to the 10x Genomics genome assembly that cost ∼$5000 USA dollars, with HudsonAlpha preparing and sequencing the linked-read library and providing bioinformatic and assembly support. Which type of platform and sequencing depth is most suited to a given project is of course contingent on project needs and budget, depending on whether the genome is a means to an end or the end goal (Chakraborty *et al.* 2016). In our case, our immediate goal was to identify QTL associated with TLB and identify SNPs markers for rapid screening of progeny in taro breeding programs, which required high sequencing accuracy.

### Genome architecture

Transposable elements (TEs) are self-replicating, mobile genomic units that are major contributors to genome diversity and size, and have key roles in chromosome structure, gene expression, and regulation (Bennetzen and Wang 2014, Orozco-Arias *et al.* 2019). Plant genomes are known for proportionally high levels of TE repeat content, for example, up to 80% of genome sequence in corn (*Zea mays*) and *Triticum urartu*, a progenitor to wheat (Ling *et al.* 2018). The TEs fall into two categories, Class I retrotransposons and Class II DNA transposons, with the former typically more ubiquitous in plants. To illustrate this point, Class I retrotransposons contributed to 21–72% of total genome sequence in five monocot cereal grains and grasses (Ling *et al.* 2018), and 30–41% of total genome sequence in *Saccharum spp*. (sugarcane, a monocot) (Garsmeur *et al.* 2018), compared to Class II transposons, which contributed < 8% of total genome sequence in each of those taxa. Our findings are consistent with the above monocot examples: the two *Araceae* genomes considered here exhibit lower and upper bounds of mobile element repeat content–20% and 80%–with retrotransposons identified as the dominant type of mobile element (a similar proportion of mobile elements went uncharacterized). The spread we observed in repeat content of Araceae is expected because of the smaller genome size of great duckweed (Michael *et al.* 2017) and the well-described variation in genome size of taro (Wang *et al.* 2017), which may be attributable to repeat content in addition to variation in chromosome numbers.

The NLR class of plant disease resistance genes show phylogenetic differences in NB-ARC domains, in addition to presence or absence of TIRs (TNL *vs.* CNL), and differ considerably in their phyletic distribution (Tarr and Alexander 2009, Yue *et al.* 2012, Steuernagel *et al.* 2015). Our findings are consistent with a low frequency of TNLs in genomes of monocotyledonous species (Cannon *et al.* 2002, Tarr and Alexander 2009): we found only 6 TNLs in taro, of which only two were complete, and only one pseudogene in duckweed. Thus, consistent with previous studies of monocots, CNLs represent a major component of the repertoire of resistance genes in taro. The number of CNLs identified for taro is consistent with numbers observed in other monocots, averaging 300 to 400, and the fewer number of CNLs in duckweed is consistent with contraction of numbers of NLRs in aquatic plants (Baggs *et al.* 2019), in conjunction with the reduced genome size of great duckweed.

### Linkage mapping and QTL analysis

Although we were not able to resolve exactly 14 linkage groups, this outcome is consistent with other studies conducted using clonally propagated heterozygous species (Nzuki *et al.* 2017, Soulard *et al.* 2017). In a study of taro, Soulard *et al.* (2017) encountered the same issue where multiple linkage groups with small number of markers (< 5) were observed. Moreover, despite Soulard *et al.*’s (2017) larger sample size of >260 taro samples per mapping population, their post-filtered number of SNP loci available for linkage mapping, 586 and 548, was similar to ours, 558. In our case, failure to resolve 14 groups could be due to many factors including a smaller sample size and use of a heterozygous reference for calling SNPs. Previously, reference-free genotyping of the ‘230’ and ‘255’ parents and the ‘1025’ population (using the same GBS data) identified 30 linkage groups using 240 SNPs (Shintaku *et al.* 2016). With a reference genome we were able to double the number of markers, however we were also able to recognize that a portion of our markers on the linkage map were ordered discordant from the order of SNPs on contigs, which in some cases were present in dense clusters (score 3, S Appendix 2). While stretches of high heterozygosity could be real–as expected given the hybridization history of taro–these also could be caused by mis-assembly, either the collapse of non-homologous reads originating from repetitive regions or chimeric joins, both of which compromise calling SNPs from short-read data. Alternatively, a minor contributor to presence of SNP clusters could arise from NLR proteins, concordant with overdominance (heterozygote advantage) in plant disease resistance genes (Michelmore and Meyers 1998). We found that some of the NLRs encompassed hypervariable genomic regions, for example, LG22 contained a complete NLR protein, 261850_nlr_1, which contained 4 SNPs across 2141 nucleotides. Another case involves LG3, with five SNPs contained in the partial NLR protein (NLR_291178), spanning 721 nucleotides. Overall, the linkage map and QTL results provide foundational resources for future work aimed at identifying functional genomic elements that underpin plant ability to resist diseases, including TLB.

In total, we found 10 QTL associated with TLB resistance. Of the 10 putative QTL identified in our study, 9 were isolate-specific with only one in common between the S1 and S3 *Phytophthora* isolates. This result is similar to other studies of *Phytophthora* host interactions where isolate-specific effects were found (Leonards-Schippers *et al.* 1994, Johnson *et al.* 2012, Truong *et al.* 2012). This phenomenon underlines the need for stacking resistant genes, because multiple races and mating types of *Phytophthora* are found in Hawaii (Shrestha *et al.* 2014).

The spread of TLB poses a major threat to taro growing regions currently free from this devastating disease. Incorporating disease resistance is the most sustainable approach to manage TLB, as research in the Pacific indicates that management measures such as chemical control are largely ineffective (Singh *et al.* 2012), although in Cameroon (Africa) the application of fungicide to taro was shown to reduce impacts from disease (Tarla *et al.* 2014). Securing taro genetic resources for future use, along with retaining culturally important types allows for the maximum flexibility in deploying new cultivars.

## Conclusions

In this study, we generated a high-quality genome assembly for taro, a root crop that is widely cultivated in tropical regions and is important for food security. Our results will inform studies of the origin, evolutionary history and breeding of this South Pacific crop. In addition, this genome may provide a framework for surveying mechanisms that underlie the formation of distinct morphological features associated with tropical tuber crops. This genome may also stimulate new genetic insights into this important tropical species. The QTLs identified from genotypes of a taro mapping population resistant to TLB can be further investigated to elucidate the genetics of that trait, with the complement of NLR sequences available as a starting point for advancing understanding of resistance system functionality. Further, the QTLs can be used to accelerate marker-assisted breeding programs. Finally, this genome project may provide a template for how to develop genomic resources in other understudied plant species.

## References

[bib1] Abbott I. A., 1992 La’au Hawai’i: traditional Hawaiian uses of plants, Bishop Museum Press, Honolulu, Hawaii.

[bib2] Armstrong E. E., Taylor R. W., Prost S., Blinston P., Van Der Meer E., 2018 Cost-effective assembly of the African wild dog (*Lycaon pictus*) genome using linked reads. Gigascience 8: 1–10. 10.1093/gigascience/giy124 PMC6350039 30346553

[bib3] Baggs, E. L., J. G. Monroe, A. Thanki, R. O’Grady, C. Schudoma *et al.*, 2020 Convergent Loss of an EDS1/PAD4 Signaling Pathway in Several Plant Lineages Reveals Co-evolved Components of Plant Immunity and Drought Response Plant Cell doi: 10.1105/tpc.19.00903 (Preprint posted May 14, 2020) 10.1105/tpc.19.00903 PMC7346574 32409319

[bib4] Bao Z., and Eddy S. R., 2002 Automated de novo identification of repeat sequence families in sequenced genomes. Genome Res. 12: 1269–1276. 10.1101/gr.88502 12176934 PMC186642

[bib5] Bellinger, M.R., 2019 SNP Calling and VCF Filtering Pipeline. protocols.io protocols.io 10.17504/protocols.io.84fhytn.

[bib6] Bennett M. D., and Leitch I. J., 1995 Nuclear DNA amounts in angiosperms. Ann. Bot. (Lond.) 76: 113–176. 10.1006/anbo.1995.1085

[bib7] Bennetzen J. L., and Wang H., 2014 The Contributions of Transposable Elements to the Structure, Function, and Evolution of Plant Genomes. Annu. Rev. Plant Biol. 65: 505–530. 10.1146/annurev-arplant-050213-035811 24579996

[bib8] Brooks F. E., 2008 Detached-leaf bioassay for evaluating taro resistance to *Phytophthora colocasiae*. Plant Dis. 92: 126–131. 10.1094/PDIS-92-1-0126 30786368

[bib9] Browning B. L., and Browning S. R., 2009 A unified approach to genotype imputation and haplotype-phase inference for large data sets of trios and unrelated individuals. Am. J. Hum. Genet. 84: 210–223. 10.1016/j.ajhg.2009.01.005 19200528 PMC2668004

[bib10] Cannon S. B., Zhu H., Baumgarten A. M., Spangler R., May G., 2002 Diversity, distribution, and ancient taxonomic relationships within the TIR and non-TIR NBS-LRR resistance gene subfamilies. J. Mol. Evol. 54: 548–562. 10.1007/s00239-001-0057-2 11956693

[bib11] Chaïr H., Traore R. E., Duval M. F., Rivallan R., Mukherjee A., 2016 Genetic diversification and dispersal of taro (*Colocasia esculenta* (l.) Schott). PLoS One 11: e0157712 10.1371/journal.pone.0157712 27314588 PMC4912093

[bib12] Chakraborty M., Baldwin-Brown J. G., Long A. D., and Emerson J. J., 2016 Contiguous and accurate de novo assembly of metazoan genomes with modest long read coverage. Nucleic Acids Res. 44: e147. 27458204 10.1093/nar/gkw654 PMC5100563

[bib13] Cho, J. J., R. A. Yamakawa, and J. Hollyer, 2007 *Hawaiian Kalo*, *Past and Future* *Sustainable Agriculture*-1, Cooperative Extension Service, CTAHR, University of Hawaii, Honolulu.

[bib14] Coates D. J., Yen D. E., and Gaffey P. M., 1988 Chromosome variation in taro, *Colocasia esculenta*. Implications for origin in the pacific. Cytologia (Tokyo) 53: 551–560. 10.1508/cytologia.53.551

[bib15] Danecek P., Auton A., Abecasis G., Albers C. A., Banks E., 2011 The variant call format and VCFtools. Bioinformatics 27: 2156–2158. 10.1093/bioinformatics/btr330 21653522 PMC3137218

[bib16] Danecek, P., S. Schiffels, and R. Durbin, 2016 Multiallelic calling model in bcftools (-m). Available at: http://samtools.github.io/ bcftools/call-m.pdf (accessed on 27 February 2019).

[bib17] Delcher A. L., Salzberg S. L., and Phillippy A. M., 2003 Using MUMmer to Identify Similar Regions in Large Sequence Sets. Curr. Protoc. Bioinformatics 1: 10–13. 10.1002/0471250953.bi1003s00 18428693

[bib18] Elshire R. J., Glaubitz J. C., Sun Q., Poland J. A., Kawamoto K., 2011 A robust, simple genotyping-by-sequencing (GBS) approach for high diversity species. PLoS One 6: e19379 10.1371/journal.pone.0019379 21573248 PMC3087801

[bib19] Food and Agriculture Organization of the United Nations, 2017 FAOSTAT statistical database. [online]. Available from: http://www.fao.org/faostat/en/ [Accessed 1 Aug 2019].

[bib20] Garsmeur O., Droc G., Antonise R., Grimwood J., Potier B., 2018 A mosaic monoploid reference sequence for the highly complex genome of sugarcane. Nat. Commun. 9: 2638 10.1038/s41467-018-05051-5 29980662 PMC6035169

[bib21] Goodwin S., Gurtowski J., Ethe-Sayers S., Deshpande P., Schatz M. C., 2015 Oxford Nanopore sequencing, hybrid error correction, and de novo assembly of a eukaryotic genome. Genome Res. 25: 1750–1756. 10.1101/gr.191395.115 26447147 PMC4617970

[bib22] Goodwin S., McPherson J. D., and McCombie W. R., 2016 Coming of age: Ten years of next-generation sequencing technologies. Nat. Rev. Genet. 17: 333–351. 10.1038/nrg.2016.49 27184599 PMC10373632

[bib23] Greenwell A. B. H., 1947 Taro: With Special Reference to Its Culture and Uses in Hawaii. Econ. Bot. 1: 276–289. 10.1007/BF02858572

[bib24] Gurevich A., Saveliev V., Vyahhi N., and Tesler G., 2013 QUAST: Quality assessment tool for genome assemblies. Bioinformatics 29: 1072–1075. 10.1093/bioinformatics/btt086 23422339 PMC3624806

[bib25] Healey A., Furtado A., Cooper T., and Henry R. J., 2014 Protocol: A simple method for extracting next-generation sequencing quality genomic DNA from recalcitrant plant species. Plant Methods 10: 21 10.1186/1746-4811-10-21 25053969 PMC4105509

[bib26] Helmkampf M., Wolfgruber T. K., Bellinger M. R., Paudel R., Kantar M. B., 2018 Phylogenetic Relationships, Breeding Implications, and Cultivation History of Hawaiian Taro (*Colocasia esculenta*) Through Genome-Wide SNP Genotyping. J. Hered. 109: 272–282. 10.1093/jhered/esx070 28992295 PMC6018804

[bib27] Herten K., Hestand M. S., Vermeesch J. R., and Van Houdt J. K. J., 2015 GBSX: A toolkit for experimental design and demultiplexing genotyping by sequencing experiments. BMC Bioinformatics 16: 73 10.1186/s12859-015-0514-3 25887893 PMC4359581

[bib28] Hubley R., Finn R. D., Clements J., Eddy S. R., Jones T. A., 2016 The Dfam database of repetitive DNA families. Nucleic Acids Res. 44: D81–D89. 10.1093/nar/gkv1272 26612867 PMC4702899

[bib29] Hulse-Kemp A. M., Maheshwari S., Stoffel K., Hill T. A., Jaffe D., 2018 Reference quality assembly of the 3.5-Gb genome of *Capsicum annuum* from a single linked-read library. Hortic. Res. 5: 4 10.1038/s41438-017-0011-0 29423234 PMC5798813

[bib30] Kongsstovu S. í., Mikalsen S. O., Homrum E., Jacobsen J. A, Flicek P., 2019 Using long and linked reads to improve an Atlantic herring (*Clupea harengus*) genome assembly. Scientific Reports 9: 17716. 31776409 10.1038/s41598-019-54151-9 PMC6881392

[bib31] Jain M., Koren S., Miga K. H., Quick J., Rand A. C., 2018 Nanopore sequencing and assembly of a human genome with ultra-long reads. Nat. Biotechnol. 36: 338–345. 10.1038/nbt.4060 29431738 PMC5889714

[bib32] Johnson E. B., Erron Haggard J., and St.Clair D. A., 2012 Fractionation, stability, and isolate-specificity of QTL for resistance to phytophthora infestans in cultivated tomato (*Solanum lycopersicum*). G3 (Bethesda) 2: 1145–1159. doi: 10.1534/g3.112.003459 23050225 10.1534/g3.112.003459 PMC3464107

[bib33] Jupe F., Pritchard L., Etherington G. J., MacKenzie K., Cock P. J. A., 2012 Identification and localisation of the NB-LRR gene family within the potato genome. BMC Genomics 13: 75 10.1186/1471-2164-13-75 22336098 PMC3297505

[bib34] Kaushal M., Sharma K. D., and Attri S., 2013 Effect of blanching on nutritional quality of dehydrated colocasia, *Colocasia esculenta* (L.) Schott leaves. Indian J. Nat. Prod. Resour. 4: 161–164.

[bib35] Koren S., Walenz B. P., Berlin K., Miller J. R., Bergman N. H., 2017 Canu: Scalable and accurate long-read assembly via adaptive κ-mer weighting and repeat separation. Genome Res. 27: 722–736. 10.1101/gr.215087.116 28298431 PMC5411767

[bib36] Kosambi D. D., 2016 The Estimation of Map Distances from Recombination Values, pp. 125–130 in D.D. Kosambi: Selected Works in Mathematics and Statistics, edited by Ramaswamy R. Springer, New Delhi 10.1007/978-81-322-3676-4_16

[bib37] Kreike C. M., Van Eck H. J., and Lebot V., 2004 Genetic diversity of taro, *Colocasia esculenta* (L.) Schott, in Southeast Asia and the Pacific. Theor. Appl. Genet. 109: 761–768. 10.1007/s00122-004-1691-z 15156282

[bib38] Kriventseva E. V., Kuznetsov D., Tegenfeldt F., Manni M., Dias R., 2019 OrthoDB v10: Sampling the diversity of animal, plant, fungal, protist, bacterial and viral genomes for evolutionary and functional annotations of orthologs. Nucleic Acids Res. 47: D807–D811. 10.1093/nar/gky1053 30395283 PMC6323947

[bib39] Kurtz S., Phillippy A., Delcher A. L., Smoot M., Shumway M., 2004 Versatile and open software for comparing large genomes. Genome Biol. 5: R12 10.1186/gb-2004-5-2-r12 14759262 PMC395750

[bib40] Langmead B., and Salzberg S. L., 2012 Fast gapped-read alignment with Bowtie 2. Nat. Methods 9: 357–359. 10.1038/nmeth.1923 22388286 PMC3322381

[bib41] Lebot V., and Aradhya K. M., 1991 Isozyme variation in taro (*Colocasia esculenta* (L.) Schott) from Asia and Oceania. Euphytica 56: 55–66.

[bib42] Lebot V., Prana M. S., Kreike N., Van Heck H., Pardales J., 2004 Characterisation of taro (*Colocasia esculenta* (L.) Schott) genetic resources in Southeast Asia and Oceania. Genet. Resour. Crop Evol. 51: 381–392. 10.1023/B:GRES.0000023453.30948.4d

[bib43] Leonards-Schippers C., Gieffers W., Schafer-Pregl R., Ritter E., Knapp S. J., 1994 Quantitative resistance to *Phytophthora infestans* in potato: A case study for QTL mapping in an allogamous plant species. Genetics 137: 66–77. 10.1093/genetics/137.1.67 PMC1205955 7914505

[bib44] Li H., Handsaker B., Wysoker A., Fennell T., Ruan J., 2009 The Sequence Alignment/Map format and SAMtools. Bioinformatics 25: 2078–2079. 10.1093/bioinformatics/btp352 19505943 PMC2723002

[bib45] Ling H. Q., Ma B., Shi X., Liu H., Dong L., 2018 Genome sequence of the progenitor of wheat A subgenome *Triticum urartu*. Nature 557: 424–428. 10.1038/s41586-018-0108-0 29743678 PMC6784869

[bib46] Ma B., Kuang L., Xin Y., and He N., 2019 New insights into long terminal repeat retrotransposons in mulberry species. Genes (Basel) 10: 285 10.3390/genes10040285 PMC6523491 30970574

[bib47] Mace E. S., Mathur P. N., Izquierdo L., Hunter D., Taylor M. B., 2006 Rationalization of taro germplasm collections in the Pacific Island region using simple sequence repeat (SSR) markers. Plant Genet. Resour. 4: 210–220. 10.1079/PGR2006125

[bib48] Margarido G. R. A., Souza A. P., and Garcia A. A. F., 2007 OneMap: Software for genetic mapping in outcrossing species. Hereditas 144: 78–79. 10.1111/j.2007.0018-0661.02000.x 17663699

[bib49] Marks P., Garcia S., Barrio A. M., Belhocine K., Bernate J., 2019 Resolving the full spectrum of human genome variation using Linked-Reads. Genome Res. 29: 635–645. 10.1101/gr.234443.118 30894395 PMC6442396

[bib50] Martinez-Viaud K. A., Lawley C. T., Vergara M. M., Ben-Zvi G., Biniashvili T., 2019 New de novo assembly of the Atlantic bottlenose dolphin (*Tursiops truncatus*) improves genome completeness and provides haplotype phasing. Gigascience 8: 1–9. 10.1093/gigascience/giy168 PMC6443575 30698692

[bib51] Matsuda M., and Nawata E., 2002 Geographical distribution of ribosomal DNA variation in taro, *Colocasia esculenta* (L.) Schott, in eastern Asia. Euphytica 128: 165–172. 10.1023/A:1020825418469

[bib52] Michael T. P., Bryant D., Gutierrez R., Borisjuk N., Chu P., 2017 Comprehensive definition of genome features in *Spirodela polyrhiza* by high-depth physical mapping and short-read DNA sequencing strategies. Plant J. 89: 617–635. 10.1111/tpj.13400 27754575

[bib53] Michelmore R. W., and Meyers B. C., 1998 Clusters of resistance genes in plants evolve by divergent selection and a birth-and-death process. Genome Res. 8: 1113–1130. 10.1101/gr.8.11.1113 9847076

[bib54] Miyasaka S. C., Bellinger M. R., Kantar M. B., Helmkampf M., Wolfgruber T. K., 2019 Genetic diversity of taro, p. 25 in Genetic Diversity in Horticultural Plants, edited by Nadwani D. Springer-Verlag, Switzerland 10.1007/978-3-319-96454-6_7

[bib55] Miyasaka S. C., McCulloch C. E., and Nelson S. C., 2012 Taro germplasm evaluated for resistance to taro leaf blight. Horttechnology 22: 838–849. 10.21273/HORTTECH.22.6.838

[bib56] Muchero W., Sewell M. M., Ranjan P., Gunter L. E., Tschaplinski T. J., 2013 Genome Anchored QTLs for Biomass Productivity in Hybrid Populus Grown under Contrasting Environments. PLoS One 8: e54468 10.1371/journal.pone.0054468 23382900 PMC3558514

[bib57] Nelson S. C., Brooks F., and Teves G., 2011 Taro Leaf Blight in Hawai’i. No. PD-71, College of Tropical Agriculture and Human Resources, University of Hawaii at Manoa, Honolulu, Hawaii.

[bib58] Nzuki I., Katari M. S., Bredeson J. V., Masumba E., Kapinga F., 2017 QTL mapping for pest and disease resistance in cassava and coincidence of some QTL with introgression regions derived from Manihot Glaziovii. Front. Plant Sci. 8: 1168 10.3389/fpls.2017.01168 28785268 PMC5519584

[bib59] Ochiai T., Nguyen V. X., Tahara M., and Yoshino H., 2001 Geographical differentiation of Asian taro, *Colacasia esculenta* (L.) Schott, detected by RAPD and isozyme analyses. Euphytica 122: 219–234. 10.1023/A:1012967922502

[bib60] Orozco-Arias S., Isaza G., and Guyot R., 2019 Retrotransposons in Plant Genomes: Structure, Identification, and Classification through Bioinformatics and Machine Learning. Int. J. Mol. Sci. 20: 3837 10.3390/ijms20153837 PMC6696364 31390781

[bib61] Ou S., and Jiang N., 2018 LTR_retriever: A highly accurate and sensitive program for identification of long terminal repeat retrotransposons. Plant Physiol. 176: 1410–1422. 10.1104/pp.17.01310 29233850 PMC5813529

[bib62] Paajanen P., Kettleborough G., López-Girona E., Giolai M., Heavens D., 2019 A critical comparison of technologies for a plant genome sequencing project. Gigascience 8: 1–12. 10.1093/gigascience/giy163 PMC6423373 30624602

[bib63] Price A. L., Jones N. C., and Pevzner P. A., 2005 De novo identification of repeat families in large genomes. Bioinformatics 21: i351–i358. 10.1093/bioinformatics/bti1018 15961478

[bib64] Rao V. R., Matthews P. J., Eyzaguirre P. B., and Hunter D., 2010 The Global Diversity of Taro: Ethnobotany and Conservation, Bioversity International, Rome, Italy.

[bib75] R Core Team, 2019 R: A language and environment for statistical computing. R Foundation for Statistical Computing, Vienna, Austria. https://www.R-project.org/.

[bib76] RStudio Team, 2015 RStudio: integrated development environment for R. RStudio, PBC, Boston, MA. http://www.rstudio.com/.

[bib65] Seppey M., Manni M., and Zdobnov E. M., 2019 BUSCO: Assessing genome assembly and annotation completeness, Methods in Molecular Biology, edited by Kollmar M. Humana, New York, NY. 10.1007/978-1-4939-9173-0_14 31020564

[bib66] Shintaku M. H., Kimball H. L., Brown A. D., Miyasaka S. C., Sim S. B., 2016 Using genotyping by sequencing (GBS) to identify loci in *Colocasiae esculenta* linked to *Phytophthora colocasiae* resistance. Acta Hortic. 1118: 131–138. 10.17660/ActaHortic.2016.1118.19

[bib67] Shrestha S., Hu J., Fryxell R. T., Mudge J., and Lamour K., 2014 SNP markers identify widely distributed clonal lineages of *Phytophthora colocasiae* in Vietnam, Hawaii and Hainan Island, China. Mycologia 106: 676–685. 10.3852/13-165 24895424

[bib68] Singh D., Jackson G., Hunter D., Fullerton R., Lebot V., 2012 Taro Leaf Blight—A Threat to Food Security. Agriculture 2: 182–203. 10.3390/agriculture2030182

[bib69] Smit A. and Hubley E., 2008-2015 RepeatModeler Open-1.0. Available online: http://www.repeatmasker.org (Accessed August 21, 2019)

[bib70] Smit A., Hubley R., and Green P., 2013-2015 RepeatMasker Open-4.0. Available online: http://www.repeatmasker.org. (Accessed August 21, 2019)

[bib71] Soulard L., Mournet P., Guitton B., and Chaïr H., 2017 Construction of two genetic linkage maps of taro using single nucleotide polymorphism and microsatellite markers. Mol. Breed. 37: 37 10.1007/s11032-017-0646-4

[bib72] Steuernagel B., Jupe F., Witek K., Jones J. D. G., and Wulff B. B. H., 2015 NLR-parser: Rapid annotation of plant NLR complements. Bioinformatics 31: 1665–1667. 10.1093/bioinformatics/btv005 25586514 PMC4426836

[bib73] Tarla D. N., Fon D. E., Takumbo E. N., and Fontem D. A., 2014 Economic evaluation of fungicide application on taro (*Colocasia esculenta*) leaf blight. J. Exp. Biol. Agric. Sci. 2: 286–292.

[bib74] Tarr D. E. K., and Alexander H. M., 2009 TIR-NBS-LRR genes are rare in monocots: Evidence from diverse monocot orders. BMC Res. Notes 2: 197 10.1186/1756-0500-2-197 19785756 PMC2763876

[bib77] Truong H. T. H., Kim K. T., Kim D. W., Kim S., Chae Y., 2012 Identification of isolate-specific resistance QTLs to phytophthora root rot using an intraspecific recombinant inbred line population of pepper (*Capsicum annuum*). Plant Pathol. 61: 48–56. 10.1111/j.1365-3059.2011.02483.x

[bib78] Wang G. Y., Zhang X. M., Qian M., Hu X. Y., and Yang Y. P., 2017 Chromosome number and genome size variation in *Colocasia* (Araceae) from China. J. Plant Res. 130: 989–997. 10.1007/s10265-017-0959-8 28642987

[bib79] Weisenfeld N. I., Kumar V., Shah P., Church D. M., and Jaffe D. B., 2017 Direct determination of diploid genome sequences. Genome Res. 27: 757–767. 10.1101/gr.214874.116 28381613 PMC5411770

[bib80] Wetzel J., Kingsford C., and Pop M., 2011 Assessing the benefits of using mate-pairs to resolve repeats in de novo short-read prokaryotic assemblies. BMC Bioinformatics 12: 95 10.1186/1471-2105-12-95 21486487 PMC3103447

[bib81] Xu L., Zhang Y., Su Y., Liu L., Yang J., 2010 Structure and evolution of full-length LTR retrotransposons in rice genome. Plant Syst. Evol. 287: 19–28. 10.1007/s00606-010-0285-2

[bib82] Xu S., 2003 Theoretical basis of the Beavis Effect. Genetics 165: 2259–2268. 14704201 10.1093/genetics/165.4.2259 PMC1462909

[bib83] Yue J. X., Meyers B. C., Chen J. Q., Tian D., and Yang S., 2012 Tracing the origin and evolutionary history of plant nucleotide-binding site-leucine-rich repeat (NBS-LRR) genes. New Phytol. 193: 1049–1063. 10.1111/j.1469-8137.2011.04006.x 22212278

[bib84] Zhang L., Meng L., Wu W., and Wang J., 2015 GACD: Integrated software for genetic analysis in clonal f1 and double cross populations. J. Hered. 106: 741–744. 26503825 10.1093/jhered/esv080

[bib85] Zheng G. X. Y., Lau B. T., Schnall-Levin M., Jarosz M., Bell J. M., 2016 Haplotyping germline and cancer genomes with high-throughput linked-read sequencing. Nat. Biotechnol. 34: 303–311. 10.1038/nbt.3432 26829319 PMC4786454

[bib86] Zimin A. V., Marçais G., Puiu D., Roberts M., Salzberg S. L., 2013 The MaSuRCA genome assembler. Bioinformatics 29: 2669–2677. 10.1093/bioinformatics/btt476 23990416 PMC3799473

